# Magnetization Dynamics Modulated by Dzyaloshinskii-Moriya Interaction in the Double-Interface Spin-Transfer Torque Magnetic Tunnel Junction

**DOI:** 10.1186/s11671-019-3150-4

**Published:** 2019-09-14

**Authors:** Simin Li, Zhaohao Wang, Yijie Wang, Mengxing Wang, Weisheng Zhao

**Affiliations:** 1 School of Microelectronics, Fert Beijing Research Institute, School of Electronics and Information Engineering, Beihang University, Beijing, 100191 China; 2 Beijing Advanced Innovation Center for Big Data and Brain Computing (BDBC), Beihang University, Beijing, 100191 China; 3 Beihang-Geortek Joint Microelectronics Institute, Qingdao Research Institute, Beihang University, Qingdao, 266100 China; 4 School of Beijing, Beihang University, Beijing, 100191 China; 5 School of Instrumentation and Optoelectronic Engineering, Beihang University, Beijing, 100191 China

**Keywords:** Magnetic tunnel junction, Spin-transfer torque, Dzyaloshinskii–Moriya interaction, Ruderman–Kittel–Kasuya–Yosida interaction

## Abstract

Currently double-interface magnetic tunnel junctions (MTJs) have been developed for enhancing the thermal stability barrier at the nanoscale technology node. Dzyaloshinskii–Moriya interaction (DMI) inevitably exists in such devices due to the use of the heavy-metal/ferromagnet structures. Previous studies have demonstrated the detrimental effect of DMI on the conventional single-interface spin-transfer torque (STT) MTJs. Here, in this work, we will prove that the detrimental effect of DMI could be almost eliminated in the double-interface STT-MTJ. This conclusion is attributed to the suppressing effect of Ruderman–Kittel–Kasuya–Yosida (RKKY) interaction on the DMI. Detailed mechanisms are analyzed based on the theoretical models and micromagnetic simulation results. Our work highlights the importance of appropriately controlling the DMI in the composite free layer of the double-interface STT-MTJ.

## Introduction

Magnetic random access memory (MRAM) is one of the most promising candidates for the next-generation non-volatile memory thanks to its low power consumption, high density, fast access speed, almost infinite endurance, and good compatibility with CMOS technology [1, 2]. The elementary device of the MRAM is the magnetic tunnel junction (MTJ), which is composed of a tunnel barrier sandwiched between two ferromagnetic layers (named pinned layer and free layer). Benefiting from the progress in the perpendicular anisotropy, the feature size of the MTJ has been scaled below 40 nm or even 1× nm [3–5]. However, a challenge for the sub-40 nm MTJ is to keep the adequate thermal stability barrier *E* = *μ*_0_*M*_*s*_*H*_*k*_*V*/2. (with *μ*_0_ the vacuum magnetic permeability, *M*_*s*_ the saturation magnetization, *H*_*k*_ the anisotropy field, *V* the volume of the free layer). As indicated by this equation, *E* decreases with the scaling of the MTJ, resulting in a reduction of data retention time. To overcome this challenge, double-interface MTJs were proposed for achieving sufficiently high *E* at the sub-40 nm technology node [6–10]. By using two coupled ferromagnetic layers as the composite free layer, the equivalent volume (*V*) in the double-interface MTJ is increased in order to enhance the thermal stability barrier. Meanwhile, the damping constant is decreased for keeping a low switching current.

In the double-interface MTJs, ferromagnet/heavy-metal (FM/HM) structure plays an important role in optimizing the performance. On the one hand, FM/HM structure increases the spin-orbit coupling (SOC) to induce the perpendicular anisotropy. On the other hand, the heavy metal works as a spacer between two ferromagnetic layers of the composite free layer to provide the Ruderman–Kittel–Kasuya–Yosida (RKKY) interaction [11], which ferromagnetically couples the magnetizations of the two ferromagnetic layers in order that they behave like an identical layer. Besides, recent works demonstrate that the strong SOC of the heavy metal combining with the atomic spins of the ferromagnet could form an antisymmetric exchange coupling called Dzyaloshinskii–Moriya interaction (DMI) [12, 13]. Therefore, the DMI is naturally induced in the double-interface MTJ with FM/HM structures. DMI favors the chiral magnetic textures (e.g., spin spirals, skyrmions, and Neel-type domain walls) and dramatically affects the magnetization dynamics, as validated by the recent studies [14–25]. It is important to mention that the role of DMI will become more complicated in the double-interface MTJ, since two FM/HM interfaces need to be considered together with an additional RKKY interaction. Therefore, it is of significance to reveal the effect of DMI on the double-interface MTJ.

In this letter, for the first time, we study the switching process of the double-interface MTJs under the actions of DMI and RKKY interaction. The double-interface MTJ is switched by the spin-transfer torque (STT), which is a mainstream approach for the data writing of the MRAM. It was recently reported that the DMI has a detrimental effect on the STT switching [21, 22]. Here, our results demonstrate that in double-interface MTJs, the detrimental effect of DMI could be suppressed by RKKY interaction, resulting in a fast switching and more uniform dynamics. Our work proves the robustness of the double-interface STT-MTJ against the negative interfacial effect.

## Methods

**Fig. 1:**
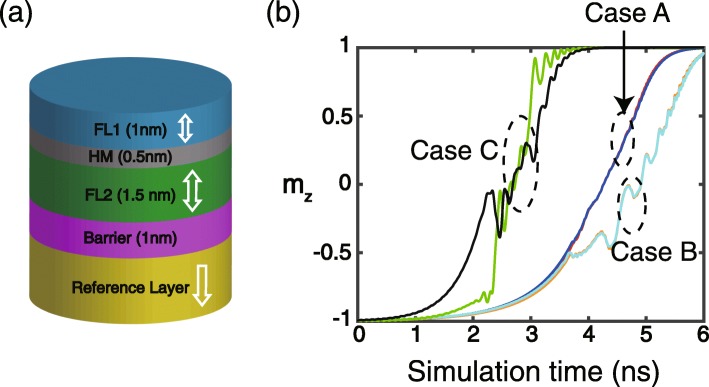
**a** Schematic structure of the device studied in this work. The other layers are not shown for clarity. **b** Typical results of the time-dependent *m*_*z*_ (perpendicular-component of the unit magnetization). Case A: σ = 1 × 10^−3^ J/m^2^, *D*_1_ = *D*_2_ = 0 (red for FL1, blue for FL2). Case B: σ = 1 × 10^−3^ J/m^2^, *D*_1_ = 1 mJ/m^2^, *D*_2_ =−1 mJ/m^2^ (orange for FL1, cyan for FL2). Case C: σ = 1 × 10^−4^ J/m^2^, *D*_1_ = *D*_2_ = 0 (green for FL1, black for FL2)

The device studied in this work is illustrated in Fig. 1a, with a FM/HM/FM structure as the composite free layer. The HM layer thickness is adjusted to an appropriate value in order that the induced RKKY interaction ferromagnetically couples two FM layers. One of the FM layers is magnetically softer, which is denoted as FL1 (free layer 1), while the other is magnetically harder and denoted as FL2 (free layer 2). To switch the magnetization of the composite free layer, a current is applied to the double-interface MTJ and generates the STT. In this work, we only consider the transmitted STT from reference layer to FL1, whereas the other torques between FL1 and FL2 are neglected. This simplified model is consistent with the previously reported works [26–28]. The DMIs are induced in both FM/HM and HM/FM interfaces and have the opposite signs due to the different chirality [29].

The magnetization dynamics of the FL1 and FL2 in the double-interface MTJ is studied by micromagnetic simulation. The time evolution of the unit magnetization vector is governed by the following Landau-Lifshitz-Gilbert (LLG) equation. We choose OOMMF package, an open-source micromagnetic simulation tool [30], to model the device structure and solve the LLG equation for analyzing the magnetization dynamics.

$$ \frac{\partial \mathbf{m}}{\partial t}=-\gamma \mathbf{m}\times {\mathbf{H}}_{eff}+\alpha \mathbf{m}\times \frac{\partial \mathbf{m}}{\partial t}+\gamma \frac{\mathrm{\hslash}}{2e}\frac{\eta }{M_s{t}_F}J\mathbf{m}\times \left(\mathbf{m}\times \mathbf{z}\right) $$

where *γ* is the gyromagnetic ratio, **m** is the unit vector along the magnetization, **z** is the unit vector along the thickness direction, **H**_eff_ is the effective field including uniaxial perpendicular anisotropy, 6-neighbor exchange energy, DMI field, RKKY interaction, demagnetization field, dipolar interaction, and STT. Other parameters and their default values are listed in Table 1, unless stated otherwise. These parameter values are in accordance with the state-of-the-art technologies. As for the DMI magnitude, we consider a CoFeB/W/CoFeB composite free layer in the double-interface MTJ [10, 31–33]. The reported experimental DMI results of W/CoFeB vary from 0.12 mJ/m^2^ to 0.73 mJ/m^2^ [34–36]. In our simulation, we extend the range of DMI magnitude to ±2 mJ/m^2^ for a general study.

**Table 1:** Parameters used in simulation

Parameters	Description	Value
*M*_*s*_	Saturation magnetization	1 MA/m
d	MTJ diameter	40 nm
α	Gilbert damping constant	0.01
P	Spin polarization	0.5
J	Applied current density	4 MA/cm^2^
A	Exchange stiffness	20 pJ/m
*K*_*u*1_	Anisotropy constant of FL1	0.8 mJ/m^3^
*K*_*u*2_	Anisotropy constant of FL2	0.7 mJ/m^3^
t_1_	Thickness of FL1	1 nm
t_2_	Thickness of FL2	1.5 nm
*D*_1_ and *D*_2_	DMI magnitudes of FL1 and FL2	− 2 to 2 mJ/m^2^
σ	Bilinear surface exchange energy for RKKY interaction	3 × 10^−4^ J/m^2^ to 10^−2^ J/m^2^

The RKKY energy between a pair of magnetic moments **m**_*i*_ and **m**_*j*_ is defined as *E*_*ij*_ = *σ*[1 − **m**_*i*_ ∙ **m**_*j*_]/*∆*_*ij*_, where **m**_*i*_ and **m**_*j*_ are magnetic moments of FL1 and FL2, respectively. *σ* is the bilinear surface exchange coefficient between two surfaces. *∆*_*ij*_ is the discretion cell size in the direction from cell *i* toward cell *j*. In this work FL1 and FL2 are ferromagnetically coupled, then *σ* > 0 which means that the RKKY interaction tends to make **m**_*i*_ parallel to **m**_*j*_. The DMI acts on the neighboring atomic spins **S**_1_ and **S**_2_ through a third atom with large SOC. Corresponding DMI Hamiltonian is expressed as *H*_*DM*_ =  − **D**_12_ ∙ (**S**_1_ × **S**_2_), where **D**_12_ is the DMI vector [37]. Therefore, the DMI degrades the uniformity between **S**_1_ and **S**_2_, which competes with the RKKY interaction.

## Results and Discussion

First of all, typical simulation results of the time-dependent *m*_*z*_ (perpendicular component of the unit magnetization) are shown in Fig. 1b. If the RKKY interaction is sufficiently strong (e.g., σ = 1 × 10^−3^J/*m*^2^ in case A and case B), FL1 and FL2 are coupled together and thus their magnetization dynamics are almost identical, no matter whether the DMI is considered or not. It is also seen that the introduction of DMI distorts the process of the magnetization switching (see case B), which is in agreement with the reported results [21–23] and can be attributed to the antisymmetric exchange of DMI. Once the RKKY interaction is not strong enough, the magnetization dynamics of FL1 and FL2 cannot be ideally coupled so that significant difference between them is observed (see case C). Below, the simulation results are obtained under a sufficiently strong RKKY interaction, unless stated otherwise.

**Fig. 2:**
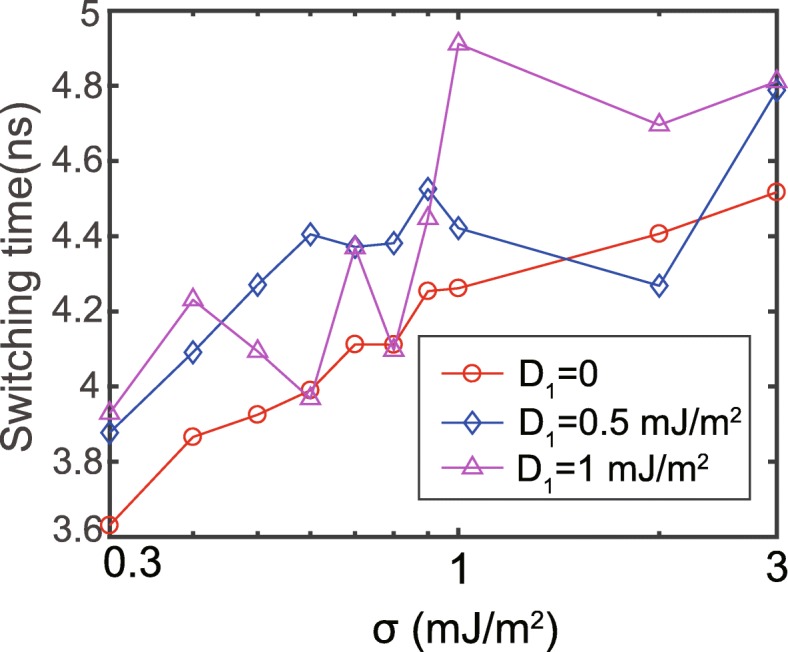
Switching time as a function of RKKY strength, with σ shown in the logarithm scale. *D*_1_ and *D*_2_ are set to the same values, but with the opposite signs

Afterwards, we study the switching speed under the various RKKY interaction. The switching speed is reflected by a time when *m*_*z*_ reaches 0 (defined as the switching time). The *D*_1_ and *D*_2_ are set to positive and negative values, respectively [29]. The corresponding results are shown in Fig. 2. In the absence of DMI, the switching time increases with the enhanced RKKY interaction, in agreement with the other reported results [26–28, 38]. The reason is that the stronger RKKY interaction makes the magnetization dynamics of FL1 and FL2 more coherently, which equivalently increases the anisotropy of the composite free layer. However, the dependence of the switching time on the RKKY strength becomes more chaotic in the presence of DMI. This chaos is mainly attributed to the inconsistency of the anisotropy between FL1 and FL2. More explanation will be shown later. These results evidence the non-negligible effect of DMI on the switching behavior of the double-interface MTJ.

**Fig. 3:**
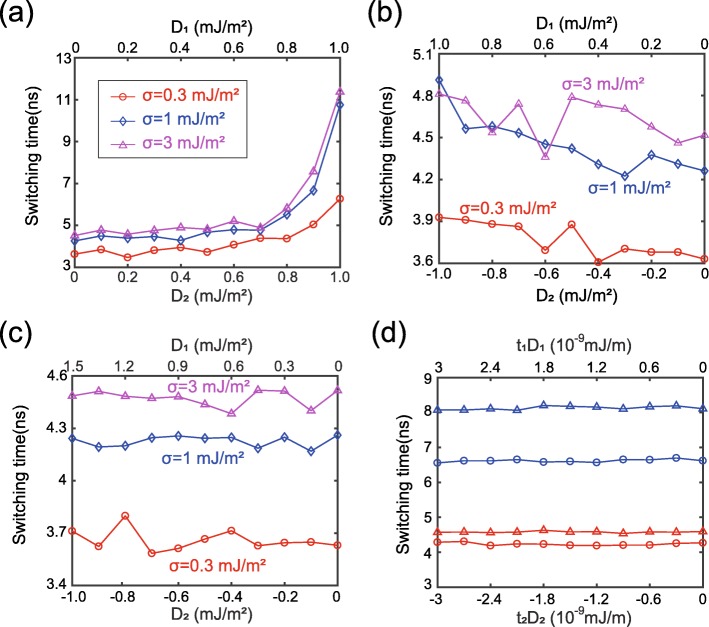
Switching time as a function of DMI strength. **a** *D*_1_ and *D*_2_ are set to the same positive value. **b** *D*_1_ and *D*_2_ are set to the same value, but with the opposite signs. **c** *D*_1_ and *D*_2_ are configured to meet *t*_1_*D*_1_ + *t*_2_*D*_2_ = 0. **d** Additional results while changing the thickness or anisotropy constant, meanwhile keeping *t*_1_*D*_1_ + *t*_2_*D*_2_ = 0. blue line: *t*_1_ is changed to 2 nm; red line: *t*_1_ is changed to 1.5 nm. Triangle data: σ = 3 × 10^−3^J/m^2^. Circle data: σ = 1 × 10^−3^J/m^2^

Next, we study the effect of DMI in more details. Figure 3 shows the switching time as a function of DMI strength. It is important to mention that *D*_1_ and *D*_2_ are intentionally set to the same positive values in Fig. 3a, although they have the opposite signs in reality. In other words, Fig. 3a corresponds to a virtual case, which we study for verifying the simulation model. From the viewpoint of physical theory, the detrimental effects of two positive DMIs are cumulated under the action of ferromagnetically coupled RKKY interaction. Therefore, the switching time is expected to rise with the increasing *D*_1_ and *D*_2_, as reported in the previous works [21, 22]. This analysis is in good agreement with the results shown in Fig. 3a. Thus, the rationality of the simulation model is validated. In contrast to Fig. 3a, the detrimental effects of DMI could be mitigated if *D*_1_ and *D*_2_ have the opposite signs, as shown in Fig. 3b, where the variation of switching time is much smaller compared with Fig. 3a. Note that in Fig. 3b, the curve is not exactly monotonous, the local fluctuation will be explained later. Remarkably, the effects of DMIs at two interfaces could be canceled out by appropriately tuning the magnitudes of *D*_1_ and *D*_2_, as shown in Fig. 3c. These results can be explained in terms of chirality theories as follows.

The DMI energy is expressed as *E*_*DM*_ = *t* ∬ *D*[*m*_*x*_(*∂m*_*z*_/*∂x*) − *m*_*z*_(*∂m*_*x*_/*∂x*) + *m*_*y*_(*∂m*_*z*_/*∂y*) − *m*_*z*_(*∂m*_*y*_/*∂y*)]*d*^2^*r* = *tDε*_*DM*_ [39], where *D* is the continuous DMI constant, *t* is the thickness of ferromagnetic layer. As mentioned above, the magnetization dynamics of FL1 and FL2 are almost identical under a sufficiently strong RKKY interaction. In this case, the same *ε*_*DM*_ is obtained in FL1 and FL2. Then the total DMI energy of FL1 and FL2 could be calculated by *E*_*tot*_ = (*t*_1_*D*_1_ + *t*_2_*D*_2_)*ε*_*DM*_. Therefore, by setting *D*_1_/*D*_2_ =  − *t*_2_/*t*_1_, the DMI effects of FL1 and FL2 could be completely offset in the case of a large enough σ, in agreement with Fig. 3c. This conclusion is further verified by the additional results shown in Fig. 3d, where the other parameters are intentionally varied meanwhile keeping *D*_1_/*D*_2_ =  − *t*_2_/*t*_1_.

**Fig. 4:**
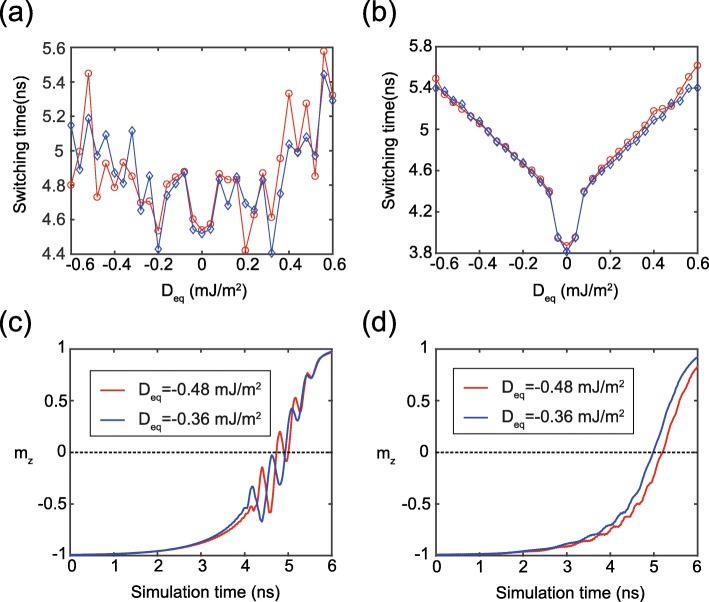
**a**, **b** Switching time as a function of *D*_eq_. Each *D*_eq_ is obtained with two pairs of different {*D*_1_, *D*_2_} values according to *D*_eq_ = (*t*_1_*D*_1_ + *t*_2_*D*_2_)/(*t*_1_ + *t*_2_). Red curve: *D*_1_ is varied meanwhile *D*_2_ is fixed to 1 mJ/m^2^. Blue curve: *D*_1_ and *D*_2_ are always set to the same value. Here σ = 1 × 10^−2^J/m^2^. In **a**, the other parameters are configured as Table 1. In **b**, *K*_*u*1_ = *K*_*u*2_ = 0.7 mJ/m^3^ for an ideal case. **c**, **d** Typical results of time-dependent *m*_*z*_ corresponding to **a** and **b**, respectively

The equivalent DMI magnitude (*D*_eq_) of the composite free layer can be expressed as *D*_eq_ = (*t*_1_*D*_1_ + *t*_2_*D*_2_)/(*t*_1_ + *t*_2_), which could be used for quantitatively analyzing the effect of DMI on the double-interface MTJ. To validate the effectiveness of this equation, we show two groups of simulation results in Fig. 4a, where two curves were obtained under the same *D*_eq_ but with two pairs of different {*D*_1_, *D*_2_} values, respectively. Although there is a little difference between the two curves, their overall trends are similar and validate the detrimental effect of DMI on the STT switching. Here, the difference between two curves could be explained as follows. FL1 and FL2 have different anisotropy constants, leading to the local uncertain oscillation of the magnetization dynamics, as shown in Fig. 4c. The same phenomenon is also observed in Fig. 2 and Fig. 3b. Instead, an ideal case is shown in Fig. 4b, d, where the anisotropy constants of FL1 and FL2 are set to the same values. Clearly, a good coincidence between the two curves is seen, indicating that the above expression of *D*_eq_ could well describe the equivalent DMI effect of the double-interface MTJ.

**Fig. 5:**
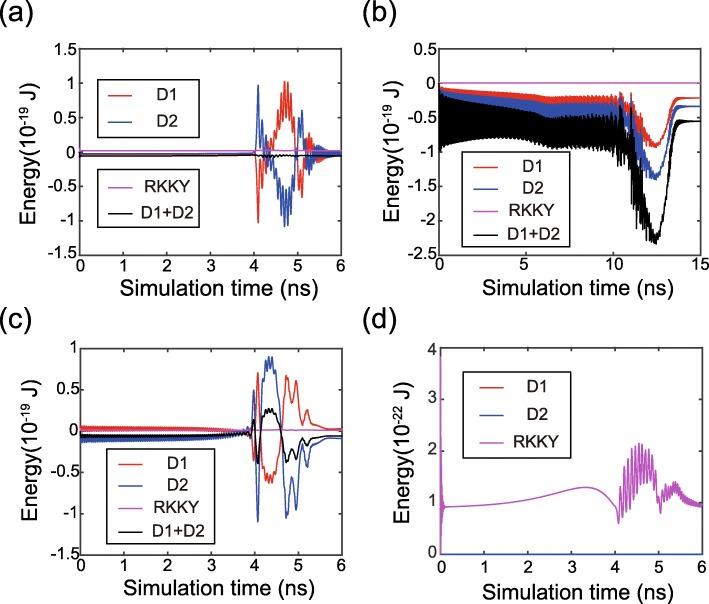
Time evolution of the DMI and RKKY energies. **a** *D*_1_ = 1.5 mJ/m^2^, *D*_2_ =  − 1 mJ/m^2^, i.e. DMI effect is canceled out. **b** *D*_1_ = *D*_2_ = 1 mJ/m^2^, i.e. DMI effect is accumulated. **c** *D*_1_ = 1 mJ/m^2^, *D*_2_ =  − 1 mJ/m^2^, i.e., DMI effect is mitigated but not canceled out. **d** *D*_1_ = *D*_2_ = 0

**Fig. 6:**
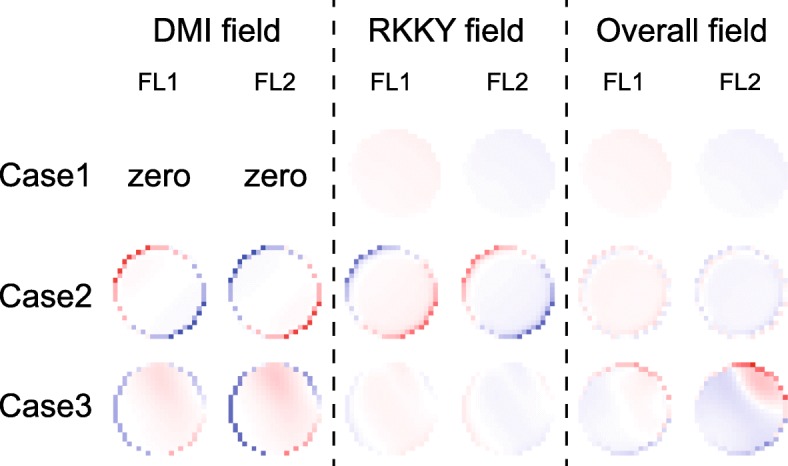
Spatial distributions of the DMI and RKKY fields. Here a typical result at one time moment is shown for each case. The conclusion remains unchanged at the other time moments. Case 1: *D*_1_ = *D*_2_ = 0. Case 2: *D*_1_ = 1.5 mJ/m^2^, *D*_2_ =  − 1 mJ/m^2^, i.e., DMI effect is canceled out. Case 3: *D*_1_ = *D*_2_ = 1 mJ/m^2^, i.e., DMI effect is accumulated

Finally, we analyze the time evolution of magnetization dynamics in more details. Figure 5 shows the time-dependent energy during magnetization switching. The DMI energies of the FL1 and FL2 are accumulated or canceled, depending on the signs and magnitudes of *D*_1_ and *D*_2_. This trend is in good agreement with the above theoretical models. In addition, the RKKY energies are kept at low values, which validates that the magnetic moments of FL1 and FL2 are synchronously driven. The distributions of RKKY and DMI fields are shown in Fig. 6, where RKKY field plays different roles in various cases. First, in the case of non-zero DMI (see case 2 and case 3), the RKKY field is much stronger compared with the case of zero DMI (see case 1). It could be understood that the RKKY field has to overcome the additional non-uniformity of the magnetic textures in the presence of DMI. Second, if *D*_1_ and *D*_2_ are of the opposite signs, the RKKY field resists the DMI fields in both FL1 and FL2 (see case 2). As a result, the DMI is weakened so that the magnetization dynamics become more uniform. In contrast, once *D*_1_ and *D*_2_ have the same sign, the RKKY field resists the DMI field in one ferromagnetic layer but assists it in the other ferromagnetic layer (see case 3). Thus the overall DMI field still has a certain effect on the magnetization dynamics, which validates that the DMI cannot be canceled out if *D*_1_ and *D*_2_ are of the same sign.

**Fig. 7:**
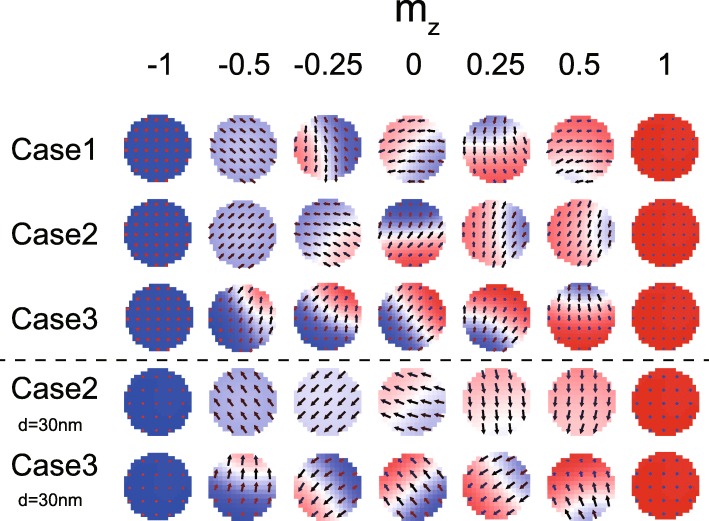
Micromagnetic configurations during the magnetization switching. Here, cases 1~3 are configured with the same parameters as Fig. 6

Figure 7 shows the micromagnetic configurations of the FL1 and FL2 during the magnetization switching. Although the domain wall appears in all the cases, different features could be observed at some time moments. It is well known that the DMI favors the non-uniform magnetic textures. Nevertheless, in Fig. 7, uniform magnetization is still formed even in the presence of DMI (see the time when *m*_*z*_ =  − 0.5 in case 2), as long as the DMI effect is canceled out. Again, this result validates the above theoretical model. In addition, it is also seen that the magnetization dynamics is more non-uniform if *D*_1_ and *D*_2_ are of the same sign (see case 3 where the domain wall always appears), consistent with the above analysis. We also show some results simulated with smaller MTJ (see the last two rows in Fig. 7). The difference of micromagnetic configurations between case 2 (DMI is canceled out) and case 3 (DMI is not canceled out) is more notable.

## Conclusion

We have comprehensively studied the effect of DMI on the double-interface STT-MTJ. As is well known, the double-interface MTJ was developed for enhancing the thermal stability barrier. In this work, our results prove another advantage of double-interface MTJ, that is, suppressing the detrimental effect of DMI. The DMIs in two ferromagnetic layers could be suppressed or even canceled out if they are configured with appropriate values and opposite signs, which is naturally satisfied by the double-interface STT-MTJ structure. Theoretical models were proposed to explain the conclusion. Micromagnetic simulation results were discussed for revealing the roles of DMI played in the magnetization dynamics. Our work provides a feasible approach to minimizing the DMI in the double-interface STT-MTJ.

## Data Availability

All data are fully available without restriction.

## Abbreviations

DMI: Dzyaloshinskii–Moriya interaction
FL: Free layer
FM/HM: Ferromagnet/heavy-metal
MRAM: Magnetic random-access memory
MTJ: Magnetic tunnel junction
RKKY: Ruderman–Kittel–Kasuya–Yosida
SOC: Spin-orbit coupling
STT: Spin-transfer torque
